# Chemerin/ChemR23 axis promotes inflammation of glomerular endothelial cells in diabetic nephropathy

**DOI:** 10.1111/jcmm.14237

**Published:** 2019-02-19

**Authors:** Jin Shang, Luyao Wang, Ya Zhang, Shiyi Zhang, Lina Ning, Jifang Zhao, Genyang Cheng, Dong Liu, Jing Xiao, Zhanzheng Zhao

**Affiliations:** ^1^ Department of Nephrology the First Affiliated Hospital of Zhengzhou University Zhengzhou China

**Keywords:** chemerin, ChemR23, diabetic nephropathy, inflammation

## Abstract

Diabetic nephropathy (DN) is characterized by inflammation of renal tissue. Glomerular endothelial cells (GEnCs) play an important role in inflammation and protein leakage in urine in DN patients. Chemerin and its receptor ChemR23 are inducers of inflammation. The aim of this study was to investigate the function of chemerin/ChemR23 in GEnCs of DN patients. Immunohistochemical staining and qRT‐PCR were used to measure the expression of chemerin, ChemR23 and inflammatory factors in renal tissues of DN patients. Db/db mice were used as animal model. ChemR23 of DN mice was knocked down by injecting LV3‐shRNA into tail vein. Inflammation, physiological and pathological changes in each group was measured. GEnCs were cultured as an in vitro model to study potential signalling pathways. Results showed that expression of chemerin, ChemR23 and inflammatory factors increased in DN patients and mice. LV3‐shRNA alleviated renal damage and inflammation in DN mice. GEnCs stimulated by glucose showed increased chemerin, ChemR23 and inflammatory factors and decreased endothelial marker CD31. Both LV3‐shRNA and SB203580 (p38 MAPK inhibitor) attenuated chemerin‐induced inflammation and injury in GEnCs. Taken together, chemerin/ChemR23 axis played an important role in endothelial injury and inflammation in DN via the p38 MAPK signalling pathway. Suppression of ChemR23 alleviated DN damage.

## 1 INTRODUCTION

Diabetic nephropathy (DN) is a severe microvascular complication of diabetes mellitus. Its prevalence continues to rise worldwide. To date, its treatment has been mainly based on the control of blood pressure and glucose. No specific treatment is available because the pathogenesis of DN is still unclear.

Innate immune system is the first line of defence against the microbial attack from the environment. However, it was revealed that inflammation was also involved in some non‐infectious chronic diseases, such as type 2 diabetes mellitus and cardiovascular disease.^1^ Persistent inflammation in the circulation system and renal tissue was an important pathophysiological basis in the development of DN.^2^ Inflammatory factors, such as interleukin (IL) −6, tumour necrosis factor (TNF) ‐α, transforming growth factor (TGF) ‐β1 and IL‐8 are involved in the development of DN. It was previously suggested that immunosuppressive reagents could significantly inhibit inflammation and even slow down or prevent the progression of DN.^3^ Multi‐glycoside of Tripterygium wilfordii Hook. f., an extract from a Chinese herbal medicine has proven to be clinically effective in relieving microinflammation in patients with early DN.^4^ However, the side effects, especially uncontrolled blood glucose or reproductive toxicity restrained the use of these treatments. As a result, to facilitate the development of effective treatments, it is necessary to understand the molecular mechanism by which inflammation drives DN.

Glomerular endothelial cells (GEnCs) form the inner layer of the glomerular filtration membrane.^5^ Long‐term exposure to high glucose (HG) will induce inflammation and injury in GEnCs.^6^ Dysfunction of GEnCs will in turn cause protein urea and renal insufficiency.^7^ However, the mechanism underling inflammation and injury in GEnCs is not been fully understood.

Chemerin, also known as tazarotene‐induced gene 2 protein or retinoid acid receptor responder 2, is a recently discovered adipocyte factor. It could be secreted by many tissues like skeletal muscles and kidney tissue besides adipose tissue. The main function of chemerin is to stimulate inflammatory factors and induce inflammatory cell infiltration. What's more, it is also believed to play a role in insulin resistance and metabolic syndrome. In type 2 diabetic patients, an elevated serum level of chemerin was associated with renal dysfunction.^8^ In streptozotocin (STZ)‐induced DN rats, chemerin was positively correlated with inflammatory factors.^9^ These results suggest that chemerin plays an important role in the development of DN by inducing renal inflammation.

There are three types of membranous receptors for chemerin, including ChemR23, CCRL2 and GPR1. However, only ChemR23 is essential for chemerin.^10^ ChemR23, or Chemokine like receptor 1, is a G protein‐coupled receptor that could direct leucocytes towards sites of inflammation.^11^ It was reported to mediate inflammation in DN rats when activated by chemerin.^12^ However, the role and molecular mechanism of chemerin/ChemR23 axis in renal damage during the development of DN is still unclear.

The aim of this study was to identify the function of chemerin/ChemR23 in GEnCs in DN patients. Patient tissue samples were collected to analyse the correlation between inflammation and chemerin/ChemR23. Db/db mice were used as a DN model to show the treatment effect of LV3‐shRNA targeting ChemR23. GEnCs were used as an in vitro model to investigate a potential signalling pathway. Our study provided evidence that the chemerin/ChemR23 axis played a key role in inflammation in DN mice and revealed a new target for DN treatment.

## 2 MATERIALS AND METHODS

### 2.1 Human sample collection

Human samples were collected from the First Affiliated Hospital of Zhengzhou University. DN samples were collected from routine renal biopsy. Included patients (n = 9) showed HG, proteinuria but normal serum creatinine. Those with diabetic complications, severe infection or accompanying diseases were excluded. Diagnosis of DN was confirmed by renal biopsy. Control group (n = 10) was set up by collecting samples from the para‐cancerous normal region of renal tumours that showed no sign of invasion under the microscope. The study was conducted according to the World Medical Association Declaration of Helsinki. Before its conduct, it was approved by the Research Ethics Committee of Zhengzhou University. Informed consent was obtained from all patients participating in the study.

### 2.2 Animal studies

C57BLKS/J background Lepr^db^/Lepr^db^ (db/db) and Lepr^db^/m (db/m) male mice were purchased from the Model Animal Research Center of Nanjing University (Nanjing, China). Db/db mice were used as a spontaneous diabetic model. They were divided into four groups: Control (db/m), DN (db/db), LV3‐NC (Lentivirus of negative control was injected into db/db mice via the tail vein at 8 weeks) and LV3‐shRNA (Lentivirus containing shRNA targeting ChemR23 was injected at the same time). All mice had unlimited access to food and water and were maintained for 20 weeks. Body weight, fasting blood glucose, serum creatinine and urinary albumin/creatinine ratio were measured to monitor the onset and progression of DN. At 16 weeks, the DN group showed significant high fasting blood glucose (>16.7 mmol/L) and protein urea. Fasting blood glucose was measured by quick sticks. Urinary protein and serum creatinine were measured by the clinical laboratory of the First Affiliated Hospital of Zhengzhou University. At the end of the study, mice were killed under deep anaesthesia. The study protocol was approved by the Institutional Animal Care and Use Committee of the First Affiliated Hospital of Zhengzhou University. The study was conducted in accordance with the National Institutes of Health (NIH) Guide for the Care and Use of Laboratory Animals.

### 2.3 Haematoxylin Eosin, Periodic acid‐Schiff, Masson and immunohistochemical staining

Haematoxylin Eosin (HE), Masson and Periodic acid‐Schiff (PAS) staining were carried out as previously described.^13^ For immunohistochemical staining, 4 μm tissue sections were incubated with the primary antibody against Collagen IV (1:100, ProteinTech Group, Chicago, IL), α‐SMA (1:100, Abcam, Cambridge, MA), TGF‐β1(1:100, Abcam, Cambridge, MA) and TNF‐α (1:100, ProteinTech Group, Chicago, IL) at 4°C overnight. Then, the sections were incubated in the secondary antibody (1:200, Goldenbridge Biotechnology Co., Beijing, China) at room temperature for 2 hours. Signal was measured using a commercial streptavidin‐biotin‐peroxidase staining kit (Goldenbridge Biotechnology Co., China).

### 2.4 Transmission electron microscopy observation

Ultra‐microstructure of the basement membrane area was observed by transmission electron microscope (TEM).^14^ Tissues for TEM were fixed in 2% glutaraldehyde/2% paraformaldehyde in 0.1 mol/L phosphate buffer, post‐fixed in buffered osmic acid, dehydrated in graded alcohols, and embedded in Epon 812 mixture. Semi‐thin sections (2 μm) were rinsed overnight in 0.1 mol/L phosphate buffer, post‐fixed for 2 hours in 1% osmium tetroxide, dehydrated and then embedded in Araldite mixture. Ultrathin sections were stained with uranyl acetate and lead citrate and examined with a CM10 TEM (Philips, Eindhoven, the Netherlands).

### 2.5 Cell culture and treatment

GEnCs were generously given by Prof. Fan Yi from Shandong University of China. Cells were maintained in RPMI‐1640 with 10% foetal bovine serum at 37°C, 5% CO2. Cells were passaged every 3‐4 days. Cells were starved for 24 hours to synchronize before stimulation. HG at a final concentration of 40 mmol/L was used as a stimulator. Mannitol at the same concentration was added as an osmolality control. Chemerin (R&D systems, Minneapolis, MN, USA) was added into the medium at 10 mmol/L or 20 mmol/L for 24 hours. p38 Mitogen‐activated protein kinase (MAPK) inhibitor SB203580 (20 μmol/L) was added into the medium 1 hour before chemerin stimulation.

### 2.6 Protein extraction and Western blot analysis

Total protein was extracted from tissue homogenate or cell lysate using RIPA buffer (CWBiotech Co., Beijing, China). Total protein was measured by the BCA protein assay kit (CWBiotech Co., Beijing, China). Western blot was performed as previously described.^15^ Sodium dodecyl sulphate polyacrylamide gel electrophoresis (SDS‐PAGE) was performed and protein was transferred to polyvinylidene fluoride membranes (Roche, Mannheim, Germany). Membranes were blocked in 3% non‐fat milk for at least 2 hours at room temperature. Then they were soaked in primary antibodies at 4°C overnight. Information about primary antibodies: anti‐chemerin (1:1000, Santa Cruz Biotechnology, Inc, Dallas, TX), anti‐ChemR23 (1:1000, Bioworld Technology, Nanjing, China), anti‐CD31 (1:1000, Abcam, Cambridge, MA), anti‐TGF‐β1 (1:1000, Abcam, Cambridge, MA), anti‐p38 (1:1000, Cell Signaling Technology, Danvers, MA), anti‐p‐p38 (1:1000, Cell Signaling Technology, Danvers, MA) and anti‐GAPDH (1:1000, Cell Signaling Technology, Danvers, MA). Membranes were washed in TBS‐T before incubation in the secondary antibody (1:1000, Dingguo Changsheng Biotech Co., Ltd., Beijing, China) at room temperature for 2 hours. Bands were measured by the enhanced chemiluminescence reagent kit (CWBiotech Co., Beijing, China).

### 2.7 RNA extraction and quantitative real‐time polymerase chain reaction

Renal tissue or cultured GEnCs were collected for total RNA extraction using TRIzol reagent (Invitrogen, Carlsbad, CA). RNA purity and concentration were determined by measuring spectrophotometric absorbance at 260 and 280 nm (A260/280). Reverse transcription of mRNA was carried out using HiScript Q RT SuperMix for qPCR (Vazyme, Nanjing, China) as previously described.^16^ Expression level of mRNA was measured by a SYBR Green PCR assay using AceQTM qPCR SYBR Green Master Mix (Novland BioPharma, Shanghai, China). GAPDH mRNA was used as an internal control for RNA normalization. Primers were synthesized by GenePharma (Shanghai, China). Mouse primers: Chemerin: F 5'‐GGAGATCGGTGTGGACAGTG‐3', R 5'‐GGGTCCAGTTTGATGCAGG‐3'; ChemR23: F 5’‐ATGGAGTACGACGCTTACAACG‐3’, R 5’‐GGTGGCGATGACAATCACCA‐3’; TGF‐β1: F 5’‐AACAACGCCATCTATGAG‐3’, R 5’‐TATTCCGTCTCCTTGGTT‐3’; CD31: F 5’‐CACAGATAAGCCCACCAGAG‐3’, R 5’‐TGACAACCACCGCAATG‐3’; GAPDH: F 5’‐TGCATCCTGCACCACCAACTGC‐3’, R 5’‐ACAGCCTTGGCAGCACCAGTGG‐3’. Human primers: chemerin: F 5’‐GAAGAAACCCGAGTGCAAA‐3’, R 5’‐ACCAACCGGCCCAGAACT‐3’; ChemR23: F 5’‐TGGTCTACAGCATCGTC‐3’, R 5’‐ATGGCTGGGGTAGGAAGAGT‐3’; TGF‐β1: F 5’‐GGTGGAAACCCACAACGAAATC‐3’, R 5’‐AATTCCCCTCCACGGCTCAAC‐3’; GAPDH: F 5’‐CACCACCAACTGCTTAG‐3’, R 5’‐CTTCACCACCTTCTTGATG‐3’. Relative mRNA expression levels were calculated using ∆ ∆ Ct analysis.

### 2.8 Gene interference

LV3‐shRNA targeting ChemR23 was synthesized by GenePharma (Shanghai, China). Specific sequence used for shRNA interference was 5’‐GCAAGATCAGCAACTTCTTGC‐3’. Multiplicity of infection (MOI) used in a cellular experiment was 100. Gene interference efficiency was measured by Western blot at 3 days after infection. In the animal study, 5 × 10^7^ TU Lentivirus was injected into the mouse tail vein.

### 2.9 Enzyme‐linked immunosorbent assay

The supernatant of cultural cells or tissue homogenate was collected. Concentrations of IL −6 (Thermo Fisher Scientific, Minneapolis, MA, USA), TGF ‐β1 (Abcam, Cambridge, MA), TNF ‐α (Abcam, Cambridge, MA) and IL‐8 (Thermo Fisher Scientific, USA) were measured by enzyme‐linked immunosorbent assay (ELISA) as previously described.^17^

### 2.10 Statistical analysis

GraphPad prism 5 (GraphPad Software Inc, San Diego, CA) was used for data analysis. Results were expressed as mean ± SD. The significance of the differences in mean values was determined using the Student's *t* test. The correlation between two parameters was tested using the Pearson analysis. *P* < 0.05 was considered to indicate a difference was statistically significant.

## 3 RESULTS

### 3.1 Expression levels of chemerin/ChemR23 in glomeruli of DN was associated with TGF‐β1

**Figure 1:**
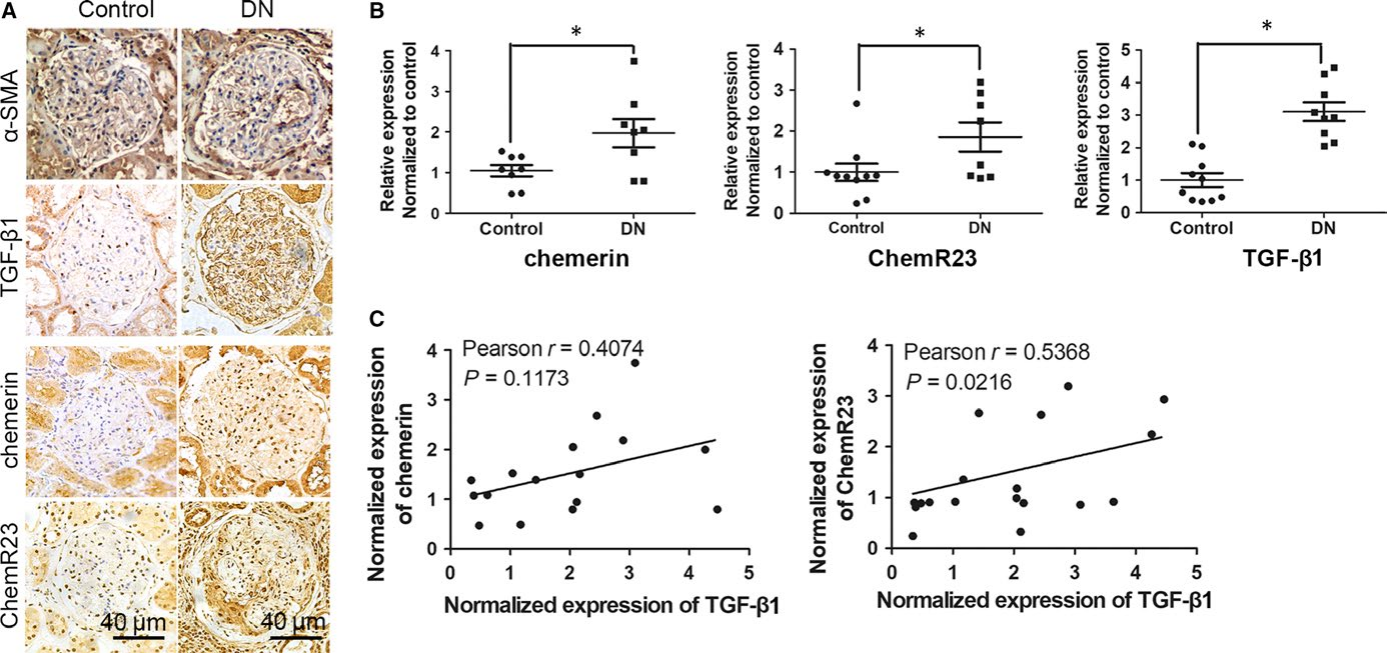
Up‐regulation of Chemerin/ChemR23 was correlated with TGF‐β1 in renal cortex of human samples. A, Expression of α‐SMA, TGF‐β1, chemerin and ChemR23 in renal cortex of DN (n = 9) and control (n = 10) samples was detected by immunohistochemical (IHC) staining. B, QRT‐PCR was used to detect the mRNA levels of chemerin, ChemR23 and TGF‐β1 in renal cortex in patient samples. GAPDH was used as an internal control. Expression was normalized to the mean value in the control group. *P < 0.05 vs control. C, Correlation analysis showed the relationship between expression of chemerin/ChemR23 and that of TGF‐β1 determined by qRT‐PCR. Pearson r and *P* values were presented in each panel

Renal cortex samples were collected from normal tissue in the para‐carcinoma region and DN patients. Results of IHC staining showed that the expression levels of chemerin, ChemR23 and TGF‐β1 in glomeruli of DN increased substantially compared with the control. Some regions in DN samples showed an increased expression level of α‐SMA in line with fibrosis. However, no differences in average expression of α‐SMA between two groups were observed (Figure 1A). Results of qRT‐PCR also showed increased expression levels of chemerin, ChemR23 and TGF‐β1 (Figure 1B). The correlation between chemerin/ChemR23 and TGF‐β1 was analysed. Results revealed a significantly positive correlation between ChemR23 and TGF‐β1 (Pearson r = 0.5368, *P* = 0.0216) and no significant correlation between chemerin and TGF‐β1 (*P* = 0.1173).

### 3.2 LV3‐shRNA was used to knock down ChemR23 of db/db mice

**Figure 2:**
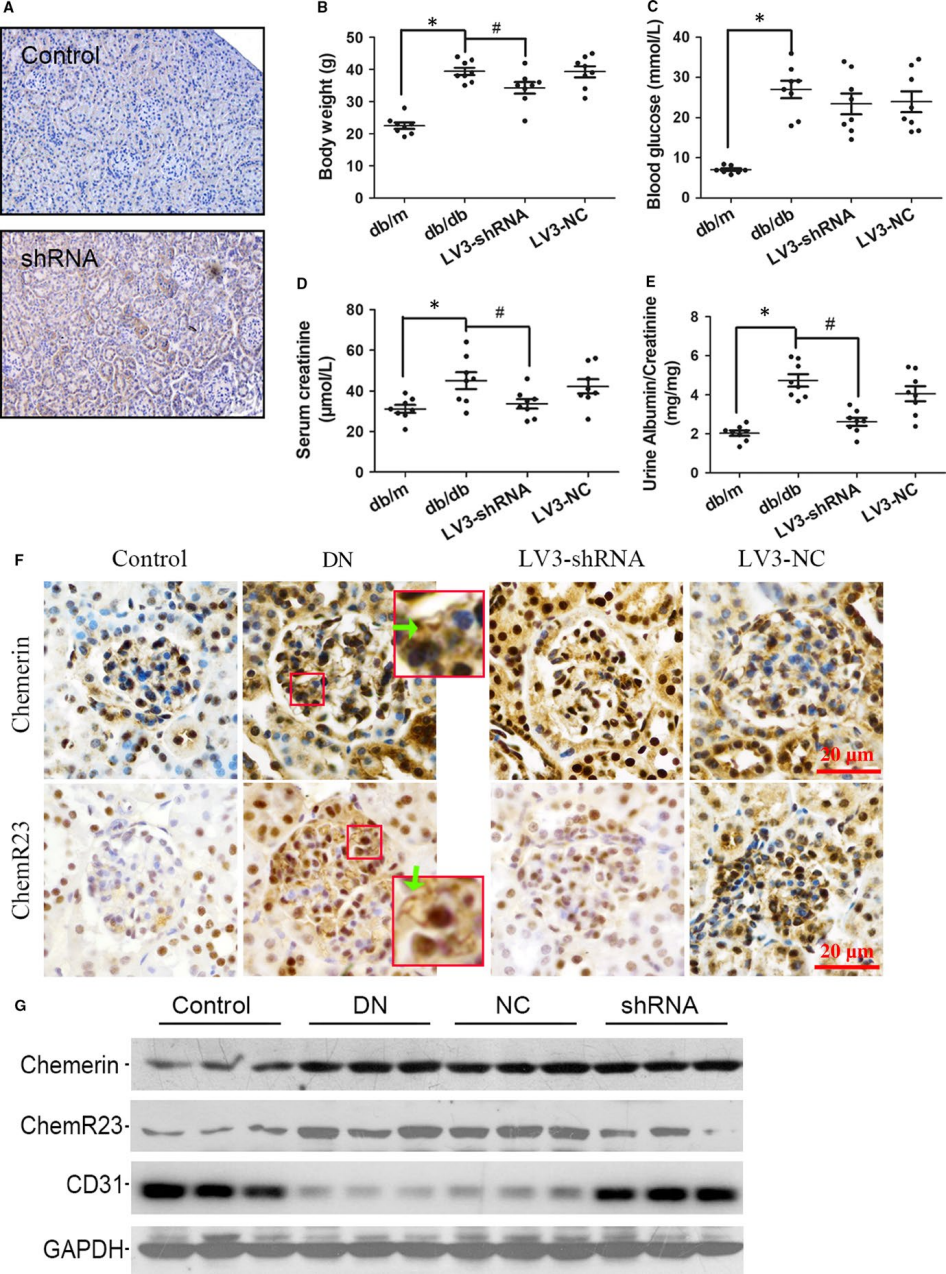
LV3‐shRNA was used to knock down ChemR23 of db/db mice. A, Immunofluorescence staining of GFP in the control or shRNA group to show the transfection efficiency. B‐E, At the end of the study, body weight, blood glucose, serum creatinine and urine albumin to creatinine ratio of mice were measured and compared among groups (n = 8). F, Representative IHC staining of chemerin and ChemR23 in each group. Green arrow shows the cytoplasmic or membrane expression. G, Western blot showed expression levels of chemerin, ChemR23 and CD31 in each group. GAPDH was used as an internal control. *P < 0.05 vs control, #*P* < 0.05 vs DN

Db/db mice were used as a spontaneous model of diabetes, while db/m were used as a control. LV3‐shRNA targeting ChemR23 or LV3‐NC (a negative control) was injected into db/db mice at 8 weeks. All mice were maintained for 20 weeks with unlimited access to food and water. Staining of GFP was shown in Figure 2A. High expression level of GFP in shRNA group indicated high transfection efficiency. Physiological changes in each group at the end of the study were shown in Figure 2B and E. Mice in db/db group showed higher levels in body weight, blood glucose, serum creatinine and urinary albumin/creatinine ratio, indicating the success of DN model. LV3‐shRNA showed a significant effect in lowering the mean value of body weight, serum creatinine and urinary albumin/creatinine ratio in DN mice (*P* < 0.05), indicating that down‐regulation of ChemR23 is effective in alleviating DN symptoms. IHC staining showed increased cytosolic expression levels of chemerin and ChemR23 in DN mice compared with the control. LV3‐shRNA could inhibit the up‐regulation of ChemR23 in DN mice. But they had no effect on chemerin (Figure 2F). Results of Western blot showed consistent changes. What's more, LV3‐shRNA could rescue the repressed expression of endothelial marker CD31 (Figure 2G). In each experiment, LV3‐NC showed no significant effect on DN mice (*P* > 0.05).

### 3.3 Down‐regulation of ChemR23 could alleviate pathological damages in DN mice

**Figure 3:**
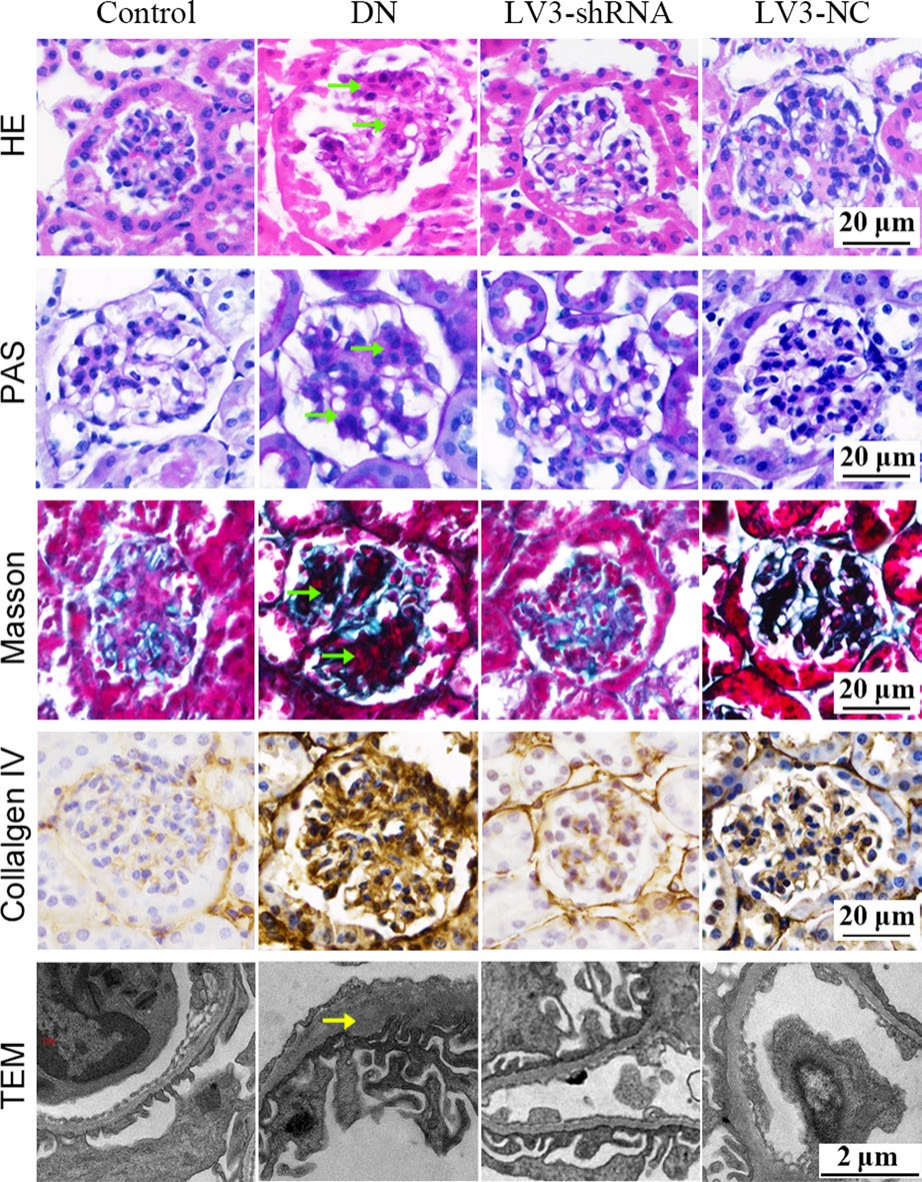
LV3‐shRNA‐ChemR23 could reverse glomerular lesions in DN mice. Representative graphs of HE, PAS and Masson staining showed typical changes in glomeruli in DN (green arrow). IHC staining showed deposition of collagen IV. TEM showed the changes in glomerular basement area (yellow arrow)

Haematoxylin Eosin, PAS and Masson staining were used to demonstrate the morphological changes in each group. In the DN group, glomeruli showed typical changes including expansion of the mesangial area, proliferation of mesangial cells, hypertrophy and fibrosis (Figure 3, green arrow). However, these changes could be substantially alleviated by LV3‐shRNA‐ChemR23. IHC staining of collagen IV showed intensive deposition in DN glomeruli while LV3‐shRNA‐ChemR23 inhibited them. TEM images in the basement area showed thickening of the basement membrane and fusion of podocyte foot processes in the DN group (yellow arrow), which was partially alleviated by LV3‐shRNA‐ChemR23. These results indicated that the DN model was successful and knock down of ChemR23 could markedly reduce glomerular damage in DN.

### 3.4 LV3‐shRNA‐ChemR23 reduced renal inflammation in DN mice

**Figure 4:**
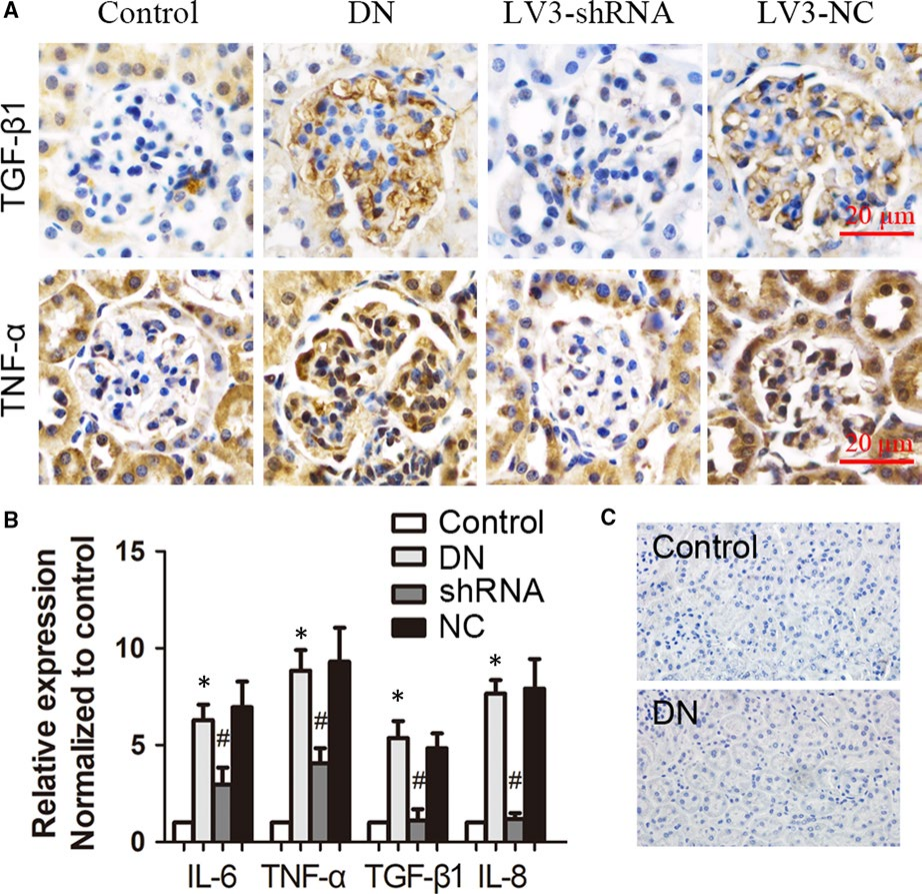
Silencing of ChemR23 alleviated micro‐inflammatory state of glomeruli in DN mice. A, Representative graphs of IHC staining showed the expression of TGF‐β1 and TNF‐α in glomeruli in DN mice. B, ELISA was used to show changes of inflammatory factors in homogenate of renal cortex. C, Immunohistochemical staining of CD68. *P < 0.05 vs control; #*P* < 0.05 vs DN (n = 3)

Expression levels of TGF‐β1 and TNF‐α in glomeruli were measured by IHC staining (Figure 4A). The homogenate of renal cortex was collected and the concentration of inflammatory factors was measured by ELISA (Figure 4B). Results indicated that DN was characterized by increased inflammatory mediators, which could be inhibited by LV3‐shRNA targeting ChemR23. CD68 is a widely used macrophage marker. In the DN group, we did not observe obvious CD68‐positive macrophages (Figure 4C).

### 3.5 Both glucose and chemerin could induce injury and inflammation of GEnCs

**Figure 5:**
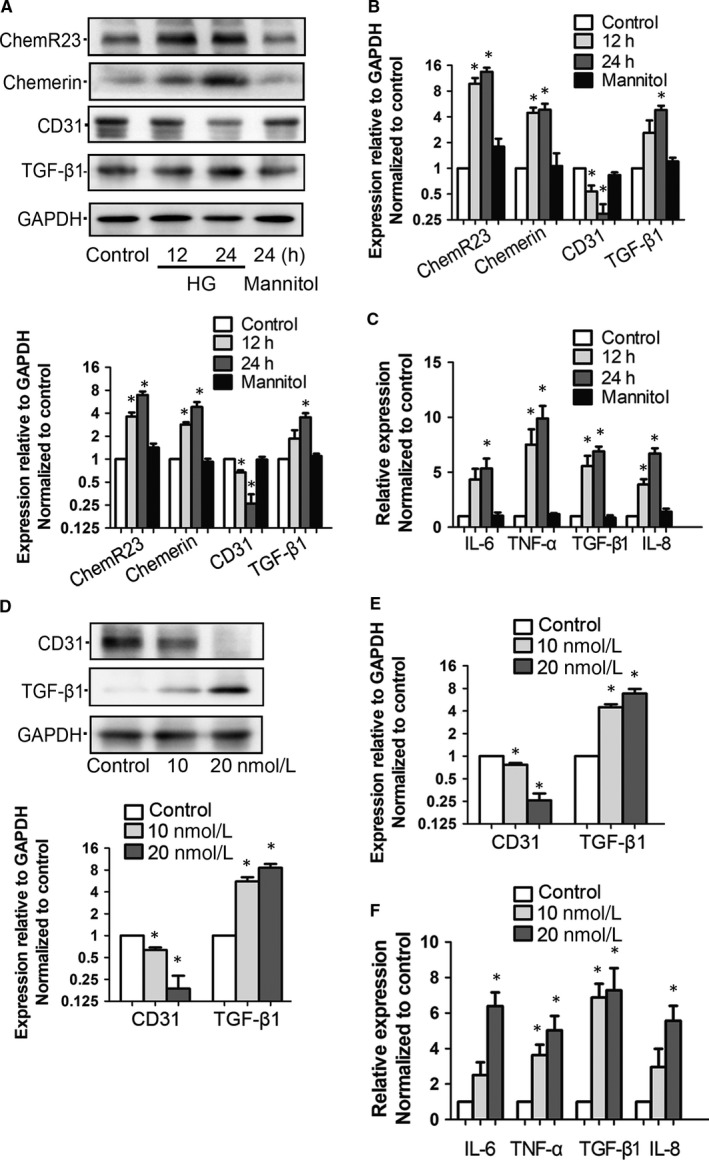
Glucose‐induced chemerin/ChemR23 could cause injury and inflammation in GEnCs. A, Representative Western blot images and summarized data showed changes of chemerin, ChemR23, CD31 and TGF‐β1 in cultured GEnCs stimulated by HG at concentration of 40 mmol/L for 12 h or 24 h. Mannitol was used as osmolality control. B, Total RNA was extracted and qRT‐PCR was used to measure the expression of mRNA of each gene in HG‐stimulated GEnCs. GAPDH was used as internal control. C, Relative concentrations of inflammatory factors in the supernatant of HG‐stimulated GEnCs (normalized to control). D, Chemerin was added into cultural medium as a stimulator at final concentration of 10 or 20 nmol/L for 24 h. Total protein was extracted and Western blot was used to show the changes of CD31 and TGF‐β1. E, qRT‐PCR showed the expression of CD31 and TGF‐β1 in chemerin‐stimulated GEnCs. F, GEnCs were stimulated by chemerin for 24 h before the supernatant was collected. ELISA was used to detect the concentrations of inflammatory factors. For all experiments, *P < 0.05 vs control (n = 3). HG, high glucose, M, mol/L

GEnCs were cultured and stimulated by HG at 40 mmol/L for 12 hours or 24 hours. Western blot showed that HG could induce the expression levels of chemerin, ChemR23 and TGF‐β1 but inhibit CD31 (Figure 5A). QRT‐PCR showed consistent results with Western blot (Figure 5B). Supernatant of HG‐stimulated GEnCs was collected and inflammatory factors were measured by ELISA. Results showed that HG was an inducer of GEnCs inflammation (Figure 5C). All results were independent of osmolality changes since mannitol (40 mmol/L, 24 hours) did not induce significant changes (*P* < 0.05). Chemerin is the ligand and activator of ChemR23. We stimulated GEnCs with chemerin at 10 or 20 nmol/L for 24 hours. Western blot and qRT‐PCR showed a decreased expression level of CD31 and increased TGF‐β1 in GEnCs (Figure 5D and E). Chemerin could also induce secretion of inflammatory factors as shown by ELISA (Figure 5F).

### 3.6 LV3‐shRNA‐ChemR23 could reverse endothelial injury and inflammation

**Figure 6:**
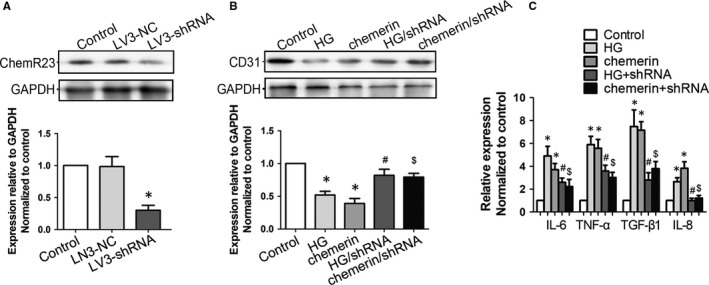
LV3‐shRNA‐ChemR23 could reverse injury and inflammation in cultured GEnCs. A, GEnCs were infected with lentivirus‐mediated shRNA targeting ChemR23 (MOI = 100). At 3 days after infection, total protein was extracted and Western blot was used to determine gene interfering efficiency. B, GEnCs were stimulated by HG (40 mmol/L) or chemerin (20 nmol/L) for 24 h. LV3‐shRNA was used to efficiently knock down ChemR23 before HG or chemerin stimulation. Western blot and summarized data showed the expression of CD31 in each group. C, Supernatant in each group was collected and inflammatory factors were measured by ELISA. *P < 0.05 vs control; #*P* < 0.05 vs HG; $*P* < 0.05 vs chemerin (n = 3)

LV3‐shRNA‐ChemR23 was used to knock down ChemR23, and Western blot confirmed high efficiency (Figure 6A). Then, we tested whether LV3‐shRNA‐ChemR23 could rescue HG or chemerin‐induced endothelial injury and inflammation. Western blot showed that reduction of CD31 was reversed by LV3‐shRNA‐ChemR23 (Figure 6B). Similarly, elevated inflammatory factors were also attenuated (Figure 6C). These results indicated that HG or chemerin‐mediated endothelial injury and inflammation were regulated by ChemR23.

### 3.7 GEnCs injury and inflammation was mediated by activation of p38 MAPK

**Figure 7:**
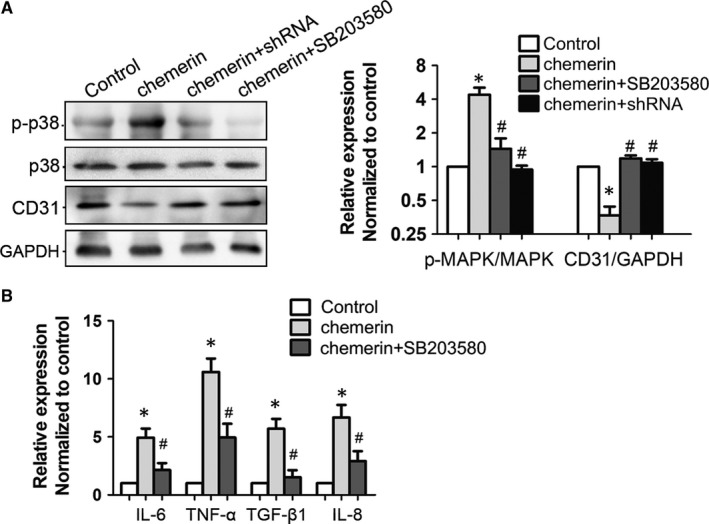
Chemerin‐induced GEnCs injury and inflammation were mediated by activation of p38 MAPK. A, GEnCs were divided into four groups: control group, chemerin‐stimulated group (20 nmol/L, 24 h), chemerin+shRNA and chemerin+SB203580 (p38 MAPK inhibitor). Activity of p38 MAKP was measured by Western blot. B, Inflammatory factors in the supernatant in each group were measured by ELISA. *P < 0.05 vs control; #*P* < 0.05 vs chemerin (n = 3)

Since chemerin was a direct activator of ChemR23, we used chemerin as a stimulator to investigate a downstream cell signalling pathway. In chemerin‐stimulated GEnCs, phosphorylated p38 MAPK increased and was inhibited by LV3‐shRNA‐ChemR23 or SB203580 (p38 MAPK inhibitor, Figure 7A). What's more, chemerin‐induced secretion of inflammatory factors was also attenuated by SB203580 (Figure 7B). These results suggested that chemerin‐induced endothelial injury and inflammation was mediated by p38 MAPK.

## 4 DISCUSSION

It is generally considered that overexpressed inflammatory factors are a promoter of renal fibrosis in different glomerular nephritis and can ultimately lead to renal failure. Many lines of evidence, ranging from in vitro experiments to epidemiological studies, have demonstrated that inflammation also plays a crucial role in the pathogenesis and progression of DN.^18^ Currently, some management targeting cytokines such as MCP‐1 have shown a promising effect in reducing urinary protein in DN.^19^ In this study, we found that chemerin could induce inflammation and cause endothelial injury in DN through its receptor ChemR23. Experiment using the DN mice model showed that gene interference targeting ChemR23 was an effective treatment to relieve renal damage. Our results added new evidence to the molecular mechanism of DN and might provide a new target for DN treatment.

Serum chemerin level was closely related with renal function in an ordinary population^20^ and incident dialysis patients.^21^ Type 2 diabetes patients with macroalbuminuria also showed significantly elevated serum levels of chemerin.^22^ DN is characterized by systematic and local microinflammation. It has been shown that the serum level of chemerin was positively correlated with the levels of inflammatory factors, including TNF‐α, IL‐6 and CRP, in patients with DN.^23^ Thus, it is reasonable to consider chemerin as a functional link between chronic inflammation and type 2 diabetes‐related disorders such as DN. A previous animal study indicated that lowering the expressing level of renal chemerin could ameliorate DN.^12^ In STZ‐induced SD rats, chemerin was clearly overexpressed in the DN group (*P* < 0.05) and correlated with inflammatory factors. Our study also showed that the expression levels of chemerin and inflammatory factors were elevated in DN patients and mice. However, the correlation between the mRNA level of chemerin and that of TGF‐β1 was not significant. The reason might be that the sample size was small, which would lead to bias. And also, since chemerin is a secretory protein, there are many other sources besides the kidney.^24^ The concentration of chemerin in renal cortex might be determined by producers other than the kidney itself. This might cover the correlation of chemerin with other factors.

Three types of chemerin receptors have been reported: ChemR23, CCRL2 and GPR1. Function of CCRL2 and GPR1 remains largely unknown. The G protein‐coupled receptor ChemR23 is a well‐defined membrane receptor for chemerin. It could lead to Gi/o‐dependent Ca2+‐mobilization when activated by chemerin.^25^ The elevated expression level of ChemR23 was reported to be associated with inflammation and renal damage in DN mice.^12^ In our study, the expression level of ChemR23 significantly increased in DN patients and was positively correlated with the TGF‐β1 expression level, indicating its relation with inflammatory mediators. Db/db mice were used as a DN model. At 20 weeks after feeding, they showed signs of DN, including significantly elevated body weight, blood glucose, serum creatinine and urinary albumin to creatinine ratio. Morphological changes in renal cortex measured by HE, PAS, Masson and TEM showed typical DN changes such as expansion of the mesangial area, proliferation of mesangial cells, thickening of GBM. IHC staining of collagen IV indicated glomerular fibrosis in DN mice. All these physical and morphological manifestations indicated that the DN mice model was successful. Expression levels of both chemerin and ChemR23 were elevated in cytosol in the DN group. Some of the nuclei were dark‐stained, and this might be due to overlap of cytosol and nucleus. Our results of immunofluorescence staining using cultured GEnCs indicated that they were mainly located in membrane and cytosol (data not shown). Blocked expression of ChemR23 significantly alleviated physical abnormalities (including body weight, serum creatinine and protein urea, *P* < 0.05) and morphological changes of glomeruli in DN. Elevated expression levels of inflammatory factors in renal cortex were also significantly attenuated by LV3‐shRNA‐ChemR23 (*P* < 0.05) in DN. All these results suggest that overexpressed ChemR23 lead to both physiological and morphological changes in DN.

The pro‐inflammatory effect of the chemerin/ChemR23 axis has been observed in different cells. Stimulating articular chondrocytes with chemerin increases the levels of TNF‐α and IL‐6 in vitro.^11^ In this study, we cultured GEnCs and then stimulated them with glucose or chemerin. Both of them could induce ChemR23 expression as well as inflammatory factors. LV3‐shRNA mediated knock down of ChemR23 and attenuated overexpressed inflammatory factors, indicating that glucose or chemerin‐induced inflammation in GEnCs via ChemR23. However, it was previously suggested that chemerin/ChemR23 also had an anti‐inflammatory effect.^26^ As a result, more attention should be focused on finding the functional balance of chemerin/ChemR23 axis‐mediated inflammation in the future. Macrophage infiltration could be observed in many kinds of inflammatory diseases. However, we did not find macrophages in renal cortex of DN mice model. Since parenchymal cells of glomeruli were reported to serve as immune cells,^27^ inflammatory factory detected in DN might be secrete by those cells instead of macrophages. It was suggested that chronic inflammation played a crucial role in the occurrence and development of DN.^28^ It has been well established that TGF‐β1 is a pro‐inflammatory factor that is involved in numerous inflammation associated diseases. In DN, elevated expression of TGF‐β1 was often accompanied by gradual fibrosis and finally led to renal failure.^29^ Our study showed that in renal biopsy samples of DN patients and renal cortex of DN mice, expression levels of TGF‐β1 and other inflammatory factors increased as ChemR23 was elevated. Since shRNA‐ChemR23 could significantly inhibit overexpression of TGF‐β1, ChemR23 might serve as a regulator of TGF‐β1. However, previous studies showed different results. In cultured mouse macrophages, TGF‐β stimulation was reported to induce overexpression of ChemR23.^30^ This, together with our results, indicates there might be a feedback loop between them. Other studies did not focus on their direct relation. However, Lin et al showed that in STZ‐induced DN rats, TGF‐β1 in serum and Chemerin in renal tissue was elevated simultaneously,^9^ suggesting a correlation between the Chemerin/ChemR23 axis and TGF‐β1. TGF‐β1 was mainly located in cytosol rather than nuclei, since Western blot using nuclear protein extraction showed very weak bands (data not shown).

As DN develops, interstitial tissue gradually shows sign of fibrosis, which is associated with chronic inflammation and finally renal failure. However, in our study, fibrosis indicator α‐SMA in DN patients did not show distinct changes. We included that this might because that DN patients had normal serum creatinine levels and mild pathological lesions. Only limited glomeruli showed signs of sclerosis. However, in the mouse model, increased serum creatinine was accompanied by glomerular deposition of collagen IV. Gene interference targeting ChemR23 showed a promising effect in inhibiting renal fibrosis possibly by reducing ChemR23‐dependent tissue inflammation.

Intact GEnCs are essential for the filtration membrane. Growing evidence indicates that dysfunction of GEnCs could aggravate renal injury in DN. In the DN mice model, inhibiting VEGF‐A could reduce endothelial activation and glomerular inflammation, therefore ultimately reversing kidney damage.^30^ HG was showed to induce renal glomerular endothelial hyperpermeability by damaging the tight junction between cells.^31^ In our study, we cultured GEnCs and then stimulated them with HG or chemerin. Results showed that chemerin/ChemR23 axis could induce inflammation and damage in GEnCs. In contrast, GEnCs could be damaged by inflammation. Endothelial injury in response to acute and subclinical inflammatory stimulation is the key step since it can cause proteinuria and renal insufficiency.^7^ This results in a vicious loop in which the chemerin/ChemR23 axis may serve as a target to stop chaos.

Mitogen‐activated protein kinase pathways include extracellular signal‐regulated kinases, c‐Jun N‐terminal kinases and p38 MAPK. p38 MAPK was reported to be involved in various cellular activities, including cell growth, differentiation, survival and death.^32^ When activated by stimulators like physiological stresses, HG or lipopolysaccharide, it could induce the production of inflammatory factors.^33^ In human endothelial cells, chemerin was reported to induce activation of the p38 MAPK pathway in a dose‐dependent manner.^34^ In the present study, we also showed that phosphorylation of p38 MAKP could be induced by chemerin. SB203580 is a widely used inhibitor of the p38 MAPK pathway. Preliminarily added of SB203580 could prevent chemerin‐induced activation of p38 MAPK. LV3‐shRNA‐ChemR23 also showed a similar effect on p38 MAPK activity, indicating p38 MAPK is the down‐stream signalling pathway of chemerin/ChemR23. In accordance, chemerin/ChemR23‐induced endothelial injury and inflammation were also blocked by either LV3‐shRNA‐ChemR23 or SB203580 suggesting involvement of p38 MAPK in these processes. The potential mechanism may be related with recruitment of G protein. When stimulated by chemerin, ChemR23 could activate G protein, which in turn phosphorylate downstream kinases and then activate signalling pathways of p38 MAPK.^35^

In summary, the main finding of this study is that chemerin/ChemR23 axis plays an important role in mediating glomerular endothelial injury and inflammation in DN via the p38 MAPK signalling pathway. Gene interference targeting ChemR23 in the mice model seems to be effective for DN treatment. Further research will be needed to validate chemerin/ChemR23 as a new strategy target for DN.

## CONFLICT OF INTEREST

The authors confirm that there are no conflicts of interest.
